# Epidemiology and clinical outcome of virus-positive respiratory samples in ventilated patients: a prospective cohort study

**DOI:** 10.1186/cc5059

**Published:** 2006-10-05

**Authors:** Cédric Daubin, Jean-Jacques Parienti, Sophie Vincent, Astrid Vabret, Damien du Cheyron, Michel Ramakers, François Freymuth, Pierre Charbonneau

**Affiliations:** 1 Department of Medical Intensive Care, Avenue Côte de Nacre, Caen University Hospital, 14033 Caen Cedex, France; 2 Department of Biostatistics and Clinical Research, Avenue Côte de Nacre, Caen University Hospital, 14033 Caen Cedex, France; 3 Inserm UMR-S 707, Université Pierre et Marie Curie-Paris6, UMR-S 707, Paris F-75012, France; 4 Department of Virology, Avenue Côte de Nacre, Caen University Hospital, 14033 Caen Cedex, France

## Abstract

**Introduction:**

Respiratory viruses are a major cause of respiratory tract infections. The prevalence of a virus-positive respiratory sample and its significance in patients requiring mechanical ventilation remain unknown.

**Methods:**

We conducted a cohort study in all consecutive adults ventilated for more than 48 hours admitted to a 22-bed medical intensive care unit during a 12-month period. Respiratory samples at the time of intubation were assessed by culture, by indirect immunofluorescence assay or by molecular methods in systematic tracheobronchial aspirates. Patients with a virus-negative respiratory sample at the time of intubation were considered unexposed and served as the control group.

**Results:**

Forty-five viruses were isolated in 41/187 (22%) patients. Rhinovirus was the most commonly isolated virus (42%), followed byherpes simplex virus type 1 (22%) and virus influenza A (16%). In multivariate analysis controlling for the Acute Pathophysiology and Chronic Health Evaluation II score, patients with respiratory disorder at admission (adjusted odds ratio, 2.1; 95% confidence interval, 0.8–5.1; *P *= 0.12), with chronic obstructive pulmonary disease/asthma patients (adjusted odds ratio, 3.0; 95% confidence interval, 1.3–6.7; *P *= 0.01) and with admission between 21 November and 21 March (adjusted odds ratio, 2.8; 95% confidence interval, 1.3–5.9; *P *= 0.008) were independently associated with a virus-positive sample. Among the 122 patients admitted with respiratory disorder, a tracheobronchial aspirate positive for respiratory viruses at the time of intubation (adjusted hazard ratio, 0.273; 95% confidence interval, 0.096–0.777; *P *< 0.006) was independently associated with better survival, controlling for the Simplified Acute Physiology Score II and admission for cardiogenic shock or cardiac arrest. Among the remaining 65 patients, a virus-positive sample on intubation did not predict survival.

**Conclusion:**

We confirmed the pathogenic role of respiratory viruses in the intensive care unit, particularly rhinovirus. We suggest, however, that the prognostic value of virus-associated respiratory disorder is better than that of other causes of respiratory disorder.

## Introduction

Respiratory viruses represent an important role in the etiology of community-acquired pneumonia in adults [1-3]. Respiratory viruses are also the leading cause of acute exacerbations of chronic obstructive pulmonary disease (COPD)/asthma patients [4,5], resulting in frequent consultations with a general practitioner and hospitalisations. In some cases, invasive ventilation is required [3,5,6]. The number of studies that document the presence of viruses in respiratory samples of critically ill patients is currently growing in the literature [7-9]. What is really needed, however, are more data on the clinical significance of these findings, particularly as regards morbidity and mortality.

In a previous work we investigated the incidence of nosocomial viral ventilator-associated pneumonia [10]. The aims of the present study were to determine the epidemiology of and risk factors for virus-positive respiratory samples taken at the time of intubation in acutely ill patients, and to compare clinical outcome (survival and time to ventilated acquired pneumonia) with and without respiratory viruses, according to the presence (group 1) or the absence (group 2) of respiratory disorder at admission.

## Methods

### Patients

All consecutively intubated adults admitted to the intensive care unit (ICU) in the University Hospital of Caen between September 2003 and September 2004 were screened, as previously reported [10].

### Data collection

Patient characteristics recorded at the time of intubation included age, sex, main reason for ICU admission, scoring of disease severity within the first day in the ICU – assessed by the admission Simplified Acute Physiology Score type II [11], the Acute Physiology and Chronic Health Evaluation II score [12] and the admission logistic organ dysfunction system [13] – and concomitant diseases such as immunocompromised status defined as HIV infection, neoplasia, innate immunity deficit, cystic fibrosis, chronic use of steroids or immunosuppressive drugs. Other comorbidities such as diabetes, COPD/asthma or cardiovascular diseases were also recorded at admission.

The main reasons for ICU admission (defined on enrolment without the knowledge of viral assessment) included cardiac arrest, septic shock, cardiac shock, mixed shock, hemorrhagic shock, respiratory distress alone (without other associated organ failure), acute renal failure, coma, intoxication, surgery and other. In addition, clinical outcomes assessed by the occurrence of ventilator-associated pneumonia and death were recorded.

According to French legislation at the time of the study and given the observational nature of our study, no ethical committee approval was requested and thus no informed consent was obtained from the patients.

### Virologic assessment

Details of the virologic methods for virus detection are published elsewhere [10]. Briefly, tracheobronchial aspirates performed at the time of intubation were assessed by culture, by indirect immunofluorescence assay or by molecular methods (PCR or RT-PCR) using previously described procedures [14-17].

The following viruses were tested: parainfluenza virus 1, parainfluenza virus 2, parainfluenza virus 3 and parainfluenza virus 4, influenza virus A, influenza virus B and influenza virus C, respiratory syncytial virus, metapneumovirus, rhinovirus, coronavirus 229E and coronavirus OC43, adenovirus, cytomegalovirus and herpes simplex virus. *Chlamydia pneumoniae *and *Mycoplasma pneumoniae *were also detected by PCR assay.

Respiratory specimens were processed for PCR or RT-PCR at the end of the study period. One positive sample and several negative control samples were included for each infectious agent, which were treated identically to the virus samples throughout. Results of conventional methods for viral isolation, routinely performed (in case of respiratory disorder), were transmitted weekly to the clinicians. Antiviral drugs could be used during the study period for proven herpes simplex virus or cytomegalovirus infection in immunocompromised patients.

### Definitions

Pneumonia was defined as any acute septic episode with respiratory symptoms (cough, sputum production, dyspnea, pleuretic chest pain or altered mental status) and a radiographic infiltrate that was neither preexisting or of other known cause [18]. Pneumonia occurring after 48 hours of hospitalisation was considered nosocomial. Ventilator-associated pneumonia was defined as described elsewhere [10]. Acute exacerbation of COPD was defined according to the NHLBI/WHO Workshop Summary [19]. Respiratory disorder was defined as respiratory distress alone or any other reasons for admission with associated respiratory symptoms.

### Statistical analysis

Quantitative data and qualitative data were expressed as the mean ± standard deviation or as the median (range) and percentage (95% confidence interval), respectively. Categorical variables were compared using the chi-square test or Fischer's exact test, when appropriate. Quantitative variables were compared using the Student *t *test or the Mann-Whitney nonparametric test, when appropriate. The confidence intervals of percentages were based on normal approximation.

We modeled the probability of a positive virus respiratory sample using a logistic regression model. Because we hypothesised that the pathogenic role of respiratory viruses in the respiratory tract may differ when associated with respiratory symptoms or not, we examined outcome according to the presence (group 1) or the absence (group 2) of respiratory disorder at admission. To assess the impact of the virus respiratory sample on time to death and time to ventilated acquired pneumonia, we constructed Kaplan-Meier curves and Cox models.

A stepwise selection of variables associated with outcome at *P *< 0.1 in the univariate analysis was chosen for multivariate modeling in both the logistic and the proportional hazards models. Multivariable modeling is a tradeoff between model complexity and parcimony. Because of our relatively small sample size, we selected a level of alpha risk <0.25 to remain in the multivariable model [20] to avoid partial confusion bias. The level of significance was set at *P *< 0.05, and all tests were two-sided.

We used EPI-INFO version 6.04dfr software (EPI-INFO; CDC, Atlanta, GA, USA) for data collection, and used EPI-INFO and SAS version 9.1 software (SAS Institute Inc., Cary, NC, USA) for data analysis.

## Results

### Prevalence and baseline characteristics

**Figure 1:**
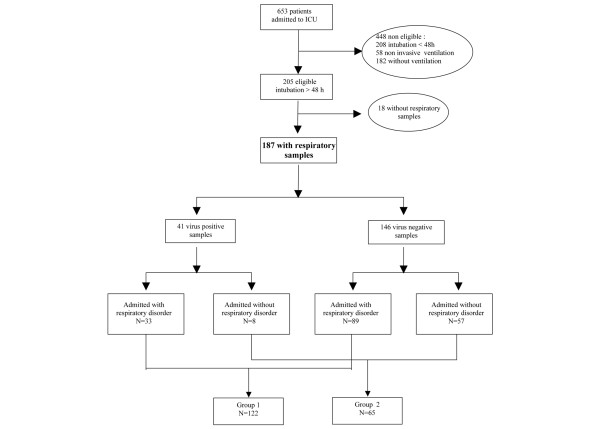
Profile of the study. ICU, intensive care unit.

Among 653 patients admitted to our ICU during the study period, a tracheobronchial aspirate was taken for viral studies in 187 patients, as shown in Figure 1. The prevalence of admitted patients with at least one virus-positive respiratory sample was 41/187 (22%; 95% confidence interval, 16–28) at the time of intubation. Baseline characteristics of patients with or without a virus-positive respiratory sample are presented in Table 1. The main reason for admission was respiratory distress alone (77/187), including 46 cases of pneumonia, nine cases of acute COPD/asthma exacerbations, 11 cases of pulmonary edema, five aspirations, two cases of intraalveolar bleedings, one case of atelectasia, one pneumothorax, one pulmonary embolism and one case of myasthenia. Forty-five out of 187 additional patients had respiratory disorders as associated symptoms at admission.

### Virus finding

**Figure 2:**
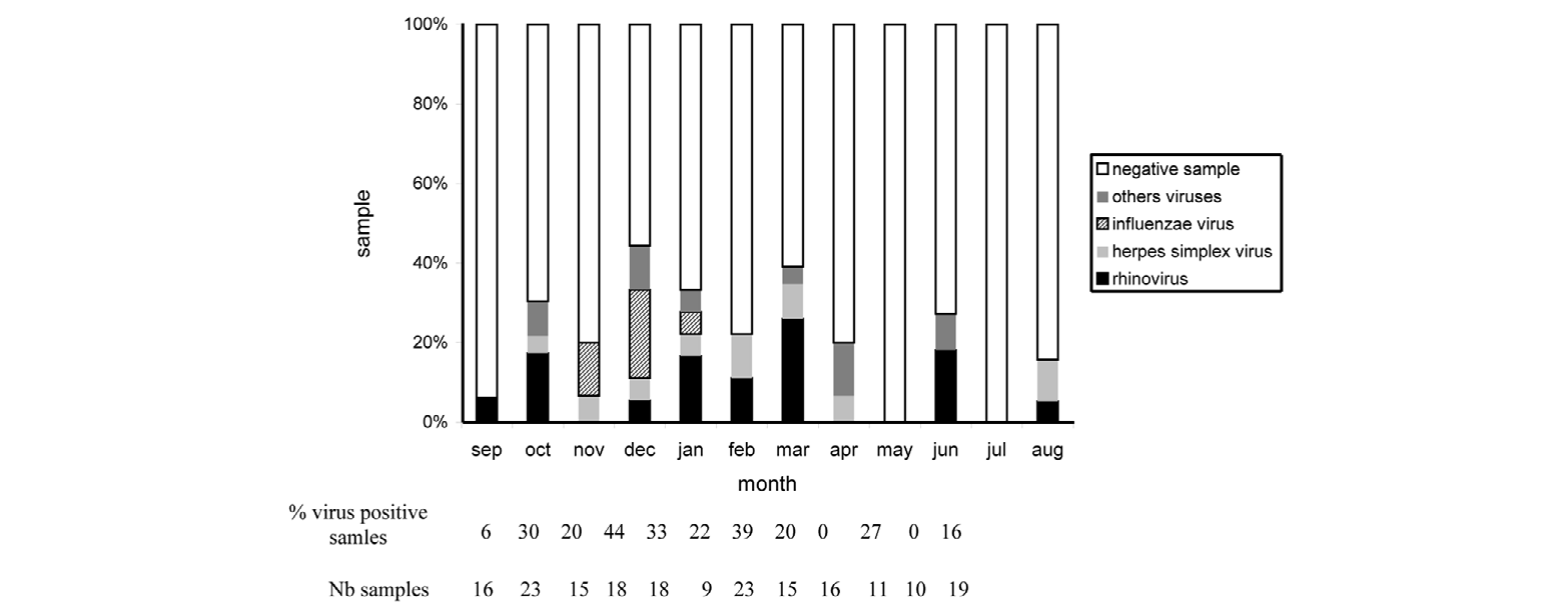
Viral endemic periods. Nb samples, number of samples.

Forty-five viruses were isolated from the respiratory specimens of 41 patients (Table 2). Rhinovirus was the most commonly isolated virus (19/45), followed by herpes simplex virus type 1 (10/45) and virus influenza A (7/45). Rhinovirus detected in the lower respiratory tract was associated with clinical signs of acute COPD exacerbation, of pneumonia or of pulmonary edema in all cases except in four patients, and virus influenza was associated with acute respiratory or cardiac failure in all cases but one. Viral coinfection was detected in four patients: one case of rhinovirus and virus parainfluenza 3, one case of rhinovirus and cytomegalovirus, one case of herpes simplex virus type 1 and virus influenza A, and one case of herpes simplex virus type 1 and coronavirus. We reported two seasonal peaks: December for virus influenza A and March for rhinovirus (Figure 2). Other viral detection details are presented in Table 2.

### Risk factors associated with a virus-positive sample

In univariate analysis (Table 1), COPD/asthma patients (*P *= 0.0012), admission between 21 November and 21 March (*P *= 0.003) and admission with respiratory disorder (*P *= 0.03) were significantly more prevalent in patients with a virus-positive sample. In addition, patients with a virus-positive sample had a nonsignificant lower Acute Physiology and Chronic Health Evaluation II score compared with patients with a virus-negative sample (18.0 versus 20.9, respectively; *P *= 0.057). In multivariate analysis controlling for Acute Physiology and Chronic Health Evaluation II score and respiratory disorder, the COPD/asthma patients and admission between 21 November and 21 March remained significantly associated with a virus-positive sample, as shown in Table 3.

### Clinical outcome by virus respiratory sample

**Figure 3:**
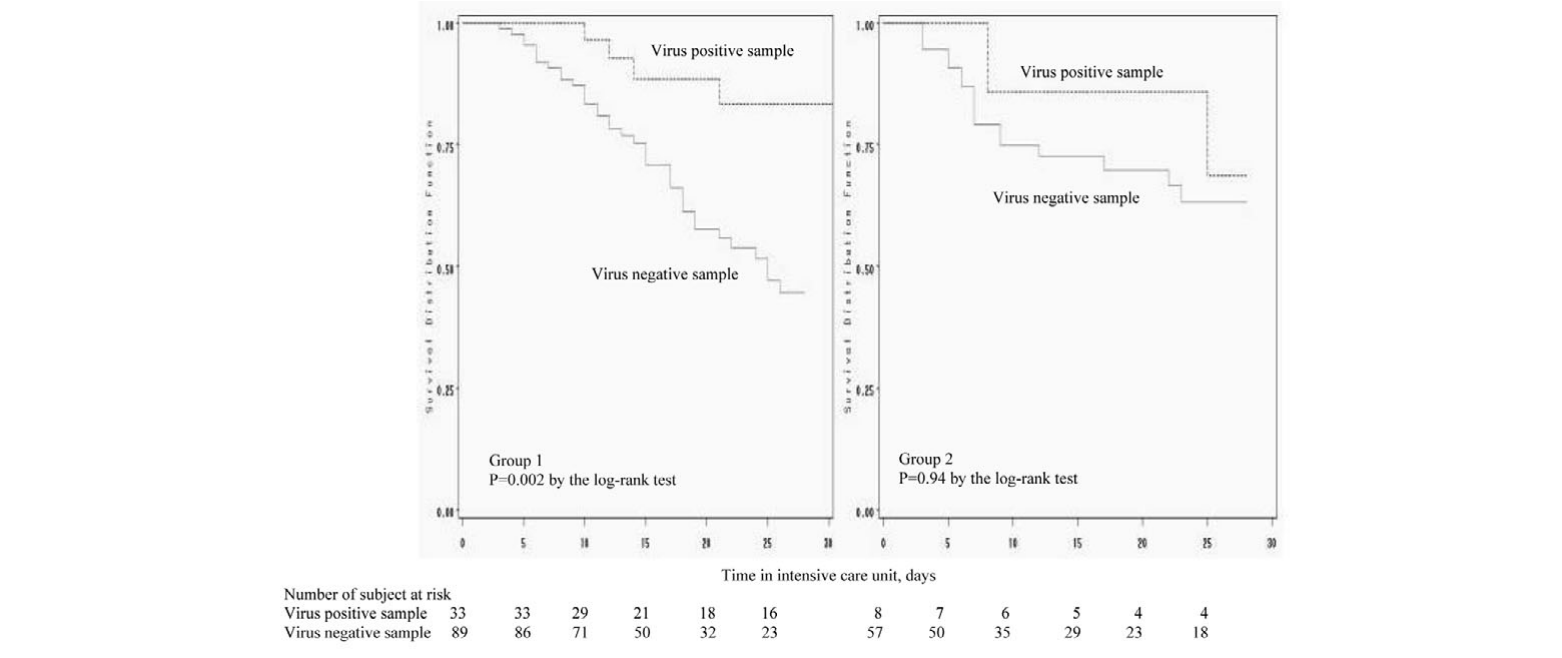
Survival according to viral screening and respiratory disorder at admission. Survival according to the result of viral screening on intubation in patients with (group 1) and without (group 2) respiratory disorder at admission.

The Kaplan-Meier curves of survival according to the presence of respiratory viruses in group 1 and in group 2 are shown in Figure 3. A tracheobronchial aspirate positive for respiratory viruses at the time of intubation was independently associated with better survival in group 1 (*P *< 0.006) but not in group 2 (*P *= 0.94), where only a higher Simplified Acute Physiology Score II (*P *< 0.002) and admission for cardiac arrest or cardiogenic shock (*P *= 0.07) predicted time to death, as shown in Table 4 and Table 5, respectively. A virus-positive sample did not predict the time to ventilator-associated pneumonia in group 1, in group 2 or overall (data not shown).

## Discussion

The present study reports that respiratory viruses, as systematically screened with sensitive methods at the time of intubation, are common (22%) among adults ventilated for more than 48 hours, regardless of the reason for admission to the ICU. Rhinovirus was the most commonly isolated virus. We have identified, for the first time in this setting, three risks factors associated with a virus-positive sample – namely, admission with respiratory disorder, COPD/asthma and admission during the winter endemic viral season. These factors highlight that the diagnosis of respiratory viral infection should be focused for patients with a respiratory disease, and support the hypothesis of the clinical impact and pathogenic role of viral infection. In addition, we suggest that the ICU mortality might be lower in viral-associated respiratory disorder than in nonviral-associated respiratory disorder. A virus-positive sample had no impact on the time to ventilator-associated pneumonia, as previously reported in a smaller sample of this cohort [10].

Our finding differs from previous studies assessing the microbiologic pattern of severe pneumonia [18,21,22] or acute exacerbation of COPD [7], which reported a lower prevalence of respiratory tract viral infection, varying from 0% [23] to 16% [7]. Differences in the diagnosis tests, the lack of a PCR assay and the limited range of viruses sought may explain this differential. Our rates of virus-positive respiratory samples were consistent with the prevalence of respiratory tract viral infections of 17–48% [8,9,24,25] observed in recent prospective studies using molecular methods for viral detection and focusing on COPD patients [9,24,25] or patients admitted to the ICU for cardiorespiratory failure [8]. As previously reported [9,26], the prevalence of virus-positive respiratory samples was increased in the endemic viral period.

The molecular method used in this study for viral detection is recognised as the most sensitive technique [27,28]. Nonetheless, the clinical relevance of a positive respiratory virus PCR test needs to be appraised. This topic has been discussed in specific populations that differed from our ventilated ICU patients; however, no chronic shedding or carriage of respiratory virus RNA was found in children [29] and no chronic shedding or carriage of respiratory syncytial virus was found in COPD patients [30]. Rhinovirus RNA could be detected up to 2–3 weeks after infection [31], without exceeding five weeks and virus influenza RNA could be detected up to seven days after infection [32]. These findings suggest that PCR-positive patients had been infected recently in our study, most of them within the two weeks before admission.

According to previous studies focusing on patients at high risk for viral disease [4,6,8,9,24,25,28,33], rhinovirus and virus influenza were the most frequently recovered viruses. These epidemiological data underscore the potential pathogenic role of rhinovirus and of influenza virus as the cause of severe respiratory disorder.

In the present study, the proportion of rhinovirus (42%) was higher than reported in ICU patients [8,9]. While its role as an important respiratory pathogen remains the subject of debate, several experimental studies with nasal inoculation demonstrated that rhinovirus could reach, penetrate and replicate in the lower airway epithelium and could induce a proinflammatory response [34,35]. Rhinovirus was also associated with severe lower respiratory tract illness [36].

In contrast, influenza virus is recognised to play a major pathogenic role during flu outbreaks in the winter-spring season. A causal relationship between influenza virus infection and hospitalisation for respiratory or cardiac failure has been shown in vaccine effectiveness studies [37,38].

We failed to demonstrate that patient exposure to respiratory viruses significantly increased the risk of ventilator-associated pneumonia. It is commonly reported that respiratory viruses could facilitate bacterial infection of the airways, by damaging the respiratory epithelium [39]. Some experimental studies have reported that respiratory viruses may promote bacterial adhesion to respiratory epithelial cells, a process that may increase bacterial colonisation [40,41], and that rhinovirus may increase the ability of *Staphylococcus aureus *to internalise into pneumocytes with a mechanism that involves the virus-induced release of IL-6 and IL-8 and the overexpression of ICAM-1 [42]. Finally, an epidemiological association has been described between viral pneumonia and nosocomial infection [43,44] or respiratory sepsis [26].

In the subgroup of patients with respiratory disorder, those with virus-positive samples surprisingly had a better survival. This result should be interpreted cautiously because it relies on the control group (that is to say, patients with a virus-negative sample). This finding does not question the severity of virus-associated respiratory disorder, but simply suggests that the prognostic may well differ from other causes of respiratory disorder. It has been reported that the clinical severity and inflammatory responses in COPD exacerbations could be modulated by the nature of the infecting organism [24,25].

We are aware of limitations. The monocenter design of the study, the relatively small number of included patients, patients' underlying disease heterogeneity as well as the fact that 18 patients (8.7%) were eligible but not screened may limit the interpretation and relevance of our data. Because the systematic search for bacteria was not obtained at the time of intubation, 'virus-associated respiratory disorder' does not necessarily mean virus-induced respiratory disorder. In addition, the PCR might be too sensitive and we cannot exclude an asymptomatic carriage of respiratory viruses in the airways in some cases. In the future, a quantifying viral load might be another approach to improve the diagnostic accuracy.

The results reported here may have important implications for the design and power analysis of a randomised controlled trial using antiviral drugs. With a 12% mortality rate in the control group (that is to say, the rate we observed in virus-associated respiratory disorder), the room for improvement in patients with viral pneumonia would be lower than that for respiratory disorder overall (34%). An appropriate sample size would consequently be necessary to demonstrate the clinical impact, if any, of antiviral drugs.

## Conclusion

We have reported and described the prevalence of virus-positive respiratory samples taken at the time of intubation in ventilated adults, contributing to improving epidemiological knowledge in the critical care setting. Using the most sensitive methods for viral detection, we were able to identify that 22% of our patients had viruses in their airways. The detection of respiratory viruses in the respiratory tract, however, was not always associated with respiratory symptoms, as demonstrated by the 12% asymptomatic carriage in group 2 (Figure 3, group 2). Finally, we suggest that patients with viruses in the respiratory tract and respiratory symptoms (suggesting a virus-associated respiratory disorder) had a better prognosis in the ICU than patients without viruses and respiratory symptoms (suggesting other causes of respiratory disorder), as shown in Figure 3 (group 1). Further studies are necessary: first, to confirm the importance of viral infections as a cause of acute respiratory failure in patients admitted to the ICU; and, second, to address the role of antiviral therapy in this population.

## Key messages

• Respiratory viruses screened at the time of intubation are common among adults ventilated for more than 48 hours, especially in patients admitted for respiratory disorder.

• Rhinovirus is the most commonly isolated virus.

• We suggest that virus-associated respiratory disorder may be associated with a lower clinical severity and better prognosis, as compared with other causes of respiratory disorder.

• Further studies are necessary to confirm the importance of viral infections as a cause of acute respiratory failure in patients admitted to the ICU, and to address the role of antiviral therapy in this patient population.

## Abbreviations

COPD = chronic obstructive pulmonary disease; ICU = intensive care unit; IL = interleukin; PCR = polymerase chain reaction; RT = reverse transcriptase.

## Competing interests

The authors declare that they have no competing interests.

## Authors' contributions

CD and SV wrote the experimental protocol and initiated the study. AV and FF performed the virologic assessments. J-JP and CD computed the statistical analysis and were involved in the interpretation of the results. CD drafted the manuscript, which was critically revised by J-JP, SV, FF, AV, DdC, MR and PC. All authors read and approved the final manuscript.

## Figures and Tables

**Table 1:** Baseline characteristics of patients with or without virus-positive respiratory samples at the time of intubation.

	Virus-positive samples (*n *= 41)	Virus-negative samples (*n *= 146)	*P *value
Age (years)	63.2 ± 16.1	62.9 ± 14.5	0.91
Male	31 (75.6)	103 (70.5)	0.53
Comorbidities			
Chronic obstructive pulmonary disease/asthma	16 (39.0)	23 (15.7)	0.0012
Cardiologic disease	37 (90.2)	124 (84.9)	0.38
Chronic use of steroids	8 (19.5)	17 (11.6)	0.19
Neoplasia	2 (4.9)	13 (8.9)	0.53
Immunosupressive drugs	2 (4.8)	6 (4.1)	1.0
Neutropenia < 1,000/mm^3^	0	2 (1.4)	1.0
Community admission	29 (70.7)	98 (67.1)	0.66
Admission between 21 November and 21 March	23 (56.1)	45 (30.8)	0.003
Reason for intensive care unit admission			
Respiratory disorder^a^	33 (75.6)	89 (61.0)	0.03
Septic shock	6 (14.6)	21 (14.4)	1.0
Cardiogenic shock or cardiac arrest	2 (4.9)	36 (24.7)	0.004
Comatose	3 (7.3)	24 (16.4)	0.2
Other	3 (7.3)	15 (10.3)	0.78
Acute Physiology and Chronic Health Evaluation type II score	18.0 ± 9.3	20.9 ± 8.3	0.057
Simplified Acute Physiology Score II	46.8 ± 18.9	51.5 ± 17.3	0.13
Logistic organ dysfunction system	6.8 ± 3.9	7.8 ± 3.8	0.14

Data are presented as the mean ± standard deviation or number (%), when appropriate. ^a^Respiratory distress alone or associated symptoms with another reason for admission.

**Table 2:** Viruses detected in the respiratory specimens on enrolment.

Patient number	COPD/asthma patient	Reason for admission	Respiratory disorder at admission	Virus detected at enrolment	Specimen tested by molecular methods	Diagnostic method	Bacteria detected at enrolment
9	Yes	Respiratory distress	COPD/asthma exacerbation	*Enterovirus*	Yes	RT-PCR	No
14	Yes	Coma	Aspiration pneumonia	Rhinovirus	Yes	RT-PCR	No
16	No	Septic shock	No	Rhinovirus	Yes	RT-PCR + culture	No
21	Yes	Respiratory distress	COPD/asthma exacerbation	Herpes simplex virus 1	No	Culture	No
30	Yes	Hemorrhagic shock	No	Virus influenza A + herpes simplex virus	Yes	RT-PCR + culture	No
31	No	Respiratory distress	Pulmonary edema	Virus influenza A	Yes	RT-PCR + immuno-fluorescence	No
37	No	Respiratory distress	Pneumonia	Rhinovirus	Yes	RT-PCR	No
39	No	Cardiac arrest	Pneumonia	Rhinovirus	Yes	RT-PCR	Hemophilus
42	No	Respiratory distress	Pneumonia	Virus influenza A	No	Immuno-fluorescence	Mycoplasma
49	No	Respiratory distress	Pneumonia	Herpes simplex virus 1	No	Culture	Pneumococcus
55	Yes	CA	Pulmonary edema	Virus influenza A	No	Immuno-fluorescence + culture	No
58	Yes	Respiratory distress	Pneumonia	Virus parainfluenza 3 + rhinovirus	Yes	Immunofluorescence + culture	Pneumoccocus + moraxella
59	Yes	Respiratory distress	Pneumonia	Virus influenza A	Yes	RT-PCR + immuno-fluorescence	No
67	No	Respiratory distress	Pneumonia	Virus influenza A	No	Immuno-fluorescence	No
72	Yes	Respiratory distress	COPD/asthma exacerbation	Respiratory syncytial virus	No	Immuno-fluorescence	No
75	Yes	Respiratory distress	Pneumonia	Virus influenza A	Yes	RT-PCR + immuno-fluorescence	No
76	No	Coma	No	Adenovirus	No	Culture	No
78	No	Septic shock	Pneumonia	Rhinovirus	Yes	RT-PCR	Legionella
81	No	Septic shock	Pneumonia	Rhinovirus	Yes	Culture	No
82	Yes	Respiratory distress	COPD/asthma exacerbation	Rhinovirus	Yes	Culture	No
85	Yes	Respiratory distress	Alveolar bleeding	Herpes simplex virus 1	Yes	Culture	No
91	No	Respiratory distress	Pneumonia	Herpes simplex virus 1	No	Culture	MSSA + serratia
94	No	Acute hepatitis	Pneumonia	Rhinovirus	Yes	RT-PCR	Hemophilus
96	No	Septic shock	Pneumonia	Coronavirus + herpes simplex virus 1	Yes	RT-PCR + culture	Pneumococcus
97	Yes	Respiratory distress	COPD/asthma exacerbation	Rhinovirus	Yes	RT-PCR	No
103	No	Respiratory distress	Pneumonia	Rhinovirus	Yes	RT-PCR	No
105	No	Weaning	Weaning	Rhinovirus	Yes	RT-PCR	No
106	No	Coma	Pulmonary edema	Rhinovirus	Yes	RT-PCR	No
115	Yes	Respiratory distress	Pneumonia	Respiratory syncytial virus	No	Immuno-fluorescence	No
118	No	Respiratory distress	Pneumonia	Rhinovirus	Yes	Culture	Pseudomonas
120	Yes	Respiratory distress	COPD/asthma exacerbation	Enterovirus	Yes	RT-PCR	No
124	No	Respiratory distress	Pneumonia	Herpes simplex virus 1	Yes	Culture	*Staphylococcus hemolyticus*
131	No	Septic shock	No	Rhinovirus	Yes	RT-PCR	No
136	No	Septic shock	Pneumonia	Herpes simplex virus 1	Yes	Culture	No
139	Yes	Respiratory distress	COPD/asthma exacerbation	Rhinovirus + cytomegalovirus	Yes	RT-PCR + Culture	No
140	No	Respiratory distress	Pneumonia	Rhinovirus	Yes	RT-PCR + culture	No
141	No	Respiratory distress	Pulmonary edema	Rhinovirus	Yes	RT-PCR	No
142	No	Respiratory distress	Pneumonia	Herpes simplex virus 1	No	Culture	*Escherichia coli*
143	No	Respiratory distress	Pneumonia	Varicella zoster virus	Yes	RT-PCR	No
144	No	Respiratory distress	Pneumonia	Herpes simplex virus 1	No	Culture	Legionella
145	Yes	Respiratory distress	COPD/asthma exacerbation	Rhinovirus	Yes	RT-PCR + culture	No

COPD, chronic obstructive pulmonary disease; MSSA, methicillin-sensitive *Staphylococcus aureus*.

**Table 3:** Risk factors for virus-positive respiratory samples at the time of intubation in 187 patients.

	Adjusted odds ratio (95% confidence interval)^a^	*P *value
Chronic obstructive pulmonary disease/asthma	3.0 (1.3–6.7)	0.01
Admission between 21 November and 21 March	2.8 (1.3–5.9)	0.008
Respiratory disorder	2.1 (0.8–5.1)	0.12
Acute Physiology and Chronic Health Evaluation type II score <20	2.0 (0.9–4.3)	0.09

^a^Multivariate stepwise logistic regression of factors associated with positive respiratory samples. Odds ratio > 1 indicates an increased probability of positive respiratory samples. c-index = 0.73

**Table 4:** Factors associated with survival in 122 patients with respiratory disorder (reason for admission or associated symptoms at admission).

Variable	Univariate Cox model		Multivariate Cox model^a^	
	
	Hazard ratio (95% confidence interval)	*P *value	Adjusted hazard ratio (95% confidence interval)	*P *value
Age	1.022 (0.995–1.050)	0.11		
Male	0.630 (0.334–1.187)	0.15		
Chronic obstructive pulmonary disease	0.619 (0.095–1.296)	0.20		
Acute Physiology and Chronic Health Evaluation type II score	1.025 (0.996–1.055)	0.093		
Simplified Acute Physiology Score II	1.015 (0.999–1.032)	0.072	1.012 (0.995–1.029)	0.16
Logistic organ dysfunction system	1.020 (0.948–1.098)	0.60		
Admission for cardiogenic shock/cardiac arrest	2.588 (1.146–5.844)	0.022	2.106 (0.924–4.802)	0.08
Virus-positive sample	0. 233 (0.083–0.653)	<0.003	0.273 (0.096–0.777)	<0.006

^a^Stepwise selection of variables associated with survival with *P *< 0.10 in the univariate analysis.

**Table 5:** Factors associated with survival in 65 patients without respiratory disorder.

Variable	Univariate Cox model		Multivariate Cox model^a^	
	
	Hazard ratio (95% confidence interval)	*P *value	Adjusted hazard ratio (95% confidence interval)	*P *value
Age	1.017 (0.986–1.049)	0.29		
Male	1.285 (0.496–3.333)	0.61		
Chronic obstructive pulmonary disease	2.228 (0.738–6.722)	0.16		
Acute Physiology and Chronic Health Evaluation type II score	1.043 (1.001–1.088)	0.047		
Simplified Acute Physiology Score II	1.035 (1.014–1.056)	<0.001	1.036 (1.014–1.059)	<0.002
Logistic organ dysfunction system	1.147 (1.037–1.269)	<0.008		
Admission for cardiogenic shock/cardiac arrest	2.462 (1.035–5.857)	0.042	2.253 (0.938–5.408)	0.07
Virus-positive sample	1.049 (0.306–3.593)	0.94		

^a^Stepwise selection of variables associated with survival with *P *< 0.10 in the univariate analysis.
